# Effect of time pressure on attentional shift and anticipatory postural control during unilateral shoulder abduction reactions in an oddball-like paradigm

**DOI:** 10.1186/1880-6805-33-17

**Published:** 2014-06-27

**Authors:** Koji Anan, Katsuo Fujiwara, Chie Yaguchi, Naoe Kiyota

**Affiliations:** 1 Department of Sports Instruction, Faculty of Sports and Human, Sapporo International University, 4-1-4-1 Kiyota, Kiyota-ku Sapporo 004-8602, Japan; 2 Department of Human Movement and Health, Graduate School of Medical Science, Kanazawa University, 13-1 Takara-machi, Kanazawa 920-8640, Japan; 3 Department of Physical Therapy, Faculty of Human Science, Hokkaido Bunkyo University, 5-196-1 Kogane-chuo, Eniwa 061-1449, Japan; 4 Department of Rehabilitation Science, Osaka Health Science University, 1-9-27 Tenma, Kita-ku, Osaka 530-0043, Japan

**Keywords:** Anticipatory postural control, Oddball-like paradigm, Unilateral shoulder abduction, Attentional shift, Event-related potentials, Electromyogram

## Abstract

**Background:**

The effect of time pressure on attentional shift and anticipatory postural control was investigated during unilateral shoulder abduction reactions in an oddball-like paradigm.

**Methods:**

A cue signal (S1) - imperative signal (S2) sequence was repeated with various S2-S1 intervals (1.0, 1.5, and 2.0 s). S2 comprised target and non-target stimuli presented at the position (9° to the left or the right) indicated by S1. Right shoulder abduction was performed only in response to target stimuli, which were presented with a 30% probability. The P1, N1, N2, and P3 components of event-related potentials were analyzed, and onset times of postural muscles (electromyographic activity of erector spinae and gluteus medius) were quantified with respect to middle deltoid activation.

**Results:**

There was no significant effect of S2-S1 interval on the latency or amplitude of P1, N1, or N2. The percentage of subjects with bimodal P3 peaks was significantly smaller and the slope of the P3 waveform in the 100 ms after the first peak was significantly steeper with a 1.0-s S2-S1 interval than with a 1.5- or 2.0-s S2-S1 interval. The onset of postural muscle activity was significantly later in the shorter interval conditions.

**Conclusions:**

These results suggest that with a shorter S2-S1 interval, that is, higher time pressure, attention was allocated to hasten the latter part of cognitive processing that may relate to attentional shift from S2 to next S1, which led to insufficient postural preparation associated with arm movement and anticipatory attention directed to S2.

## Background

When performing a rapid arm flexion while standing, postural muscles of the legs and trunk are activated before the focal muscles of the arm to moderate the postural disturbance caused by the movement [1,2]. When a reaction task that involves arm flexion is repeatedly performed with shortening of the interval between repetitions, that is, increasing time pressure, the speed of behavior and cognitive processing (including attentional shift) needs to increase.

The onset time of postural muscle activity with respect to the focal muscle of arm flexion is later in a simple-reaction task than in a choice-reaction task [3-5]. In these studies, there was no limitation of the response start time; therefore, time pressure was highest in the simple-reaction task, in which the speed of the response was regarded as important, and relatively lower in the choice-reaction task, in which the accuracy of stimulus judgment was most important. Thus, in the simple-reaction task, the effect of time pressure would be clearly reflected in the reaction time. However, it is difficult that with the onset time of postural muscles in these reaction tasks, we separately evaluated the effect of time pressure on the behavior and cognitive processing.

An oddball task is widely used to investigate the cognitive processing [6]. This is a choice-reaction task in which low frequent target stimuli appear repeatedly in a random order with frequent non-target stimuli, and participants are required to perform a response to the target stimuli, mostly a finger flexion. In this task, the sensory, perceptual, and cognitive processing of the stimuli can be evaluated using various components of event-related potentials (ERPs). The P1 and N1 components of ERPs are recorded from occipital electrodes contralateral to the side of the visual field in which stimuli are presented and reflect visual-sensory processing in the extrastriate visual cortex [7,8]. The N2 component is the occipital negativity with a latency of approximately 200 ms after the target stimulus, and reflects the discrimination of task-relevant visual features [9,10]. The P3 component is the parietocentral positivity with a latency of approximately 300 ms after the target stimulus, and reflects cognitive processing, such as the evaluation and judgment of a sensory stimulus [11] and subsequent context updating [12,13]. In a Stroop paradigm with difficult discrimination of target stimuli [14] and a choice-reaction task with multiple alternatives [15], P3 tended to have bimodal peaks. This suggests that P3 would have bimodal peaks when it takes a long time to evaluate and judge the stimuli, and that the second peak might be related to the latter part of this cognitive processing. This interpretation is supported by studies that used intracranial recordings, lesion studies, and functional magnetic resonance imaging to investigate the generator of P3 [16]. In a transient choice-reaction task using a finger-movement response, in which time pressure was manipulated by limiting the time available for response execution, P3 amplitude increased as time pressure increased, and the second P3 peak began to overlap with the first peak [17]. In this study, it can be observed that the slope of the P3 waveform after the first peak became steeper as the time pressure increased [17]. Thus, the amplitude of the first P3 peak and the slope after that peak can be considered as indices of the latter part of cognitive processing, including context updating.

P3 amplitude varies according not only to the inter-stimulus interval of target stimuli, but also to the probability of target stimulus presentation in the oddball paradigm [18]. Thus, a visual oddball-like paradigm incorporating a cue signal (S1) and an imperative signal (S2) [7,19] in which S1 indicates the position of the subsequent S2 can be used to manipulate time pressure. Performance of this task requires careful attention to be paid to S1. The interval from S2 to the next S1 determines the time pressure on the attentional shift from S2 to S1, when the S2-S1 interval is fixed and predictable. This method can therefore be used to investigate the changes in the P3 waveform that are caused by the time pressure.

Motor preparation, which is a part of behavior processing, and anticipatory attention directed to S2 have been evaluated using contingent negative variation (CNV) [20,21]. If the S2-S1 interval changes, CNV also changes. However, with a short S2-S1 interval, it is likely that the arm movement response to target S2 stimuli will overlap with the cognitive processing of the subsequent S1. Therefore, motor preparation before S2 and anticipatory attention directed to S2 cannot be analyzed using CNV [20]. CNV is significantly correlated with the onset time of postural muscle activation relative to the onset of focal muscle activation [22,23]. Thus, the onset time of postural muscle activation may also reflect postural preparation associated with arm movement and anticipatory attention directed to S2. We hypothesize that with greater time pressure, the attentional shift from S2 to S1 will be hastened and the postural preparation and anticipatory attention directed to S2 will be insufficient, resulting in a later onset of postural muscle activation.

In the present study, we manipulated the S2-S1 interval in an oddball-like paradigm with repeated unilateral shoulder abduction reactions, and investigated the effect of time pressure on the attentional shift and onset time of postural muscle activation. Our working hypotheses were as follows. As S2-S1 interval decreased: (1) P3 would have a unimodal peak, the amplitude of the first P3 peak would increase and the slope after the first P3 peak would become steeper; and (2) the onset of postural muscle activation would be later with respect to the onset of focal muscle activation.

## Methods

### Subjects

Subjects were 13 right-handed men. Mean (standard deviation (SD)) age, height, weight, and foot length were 21.1 (3.4) years, 174.0 (5.3) cm, 68.0 (8.4) kg, and 25.5 (0.5) cm, respectively. All subjects had normal or corrected-to-normal vision. No subject had any history of neurological or orthopedic impairment. In accordance with the Declaration of Helsinki, all subjects provided informed consent after receiving an explanation of the experimental protocol, which was approved by our Institutional Ethics Committee.

### Apparatus and data recording

**Figure 1:**
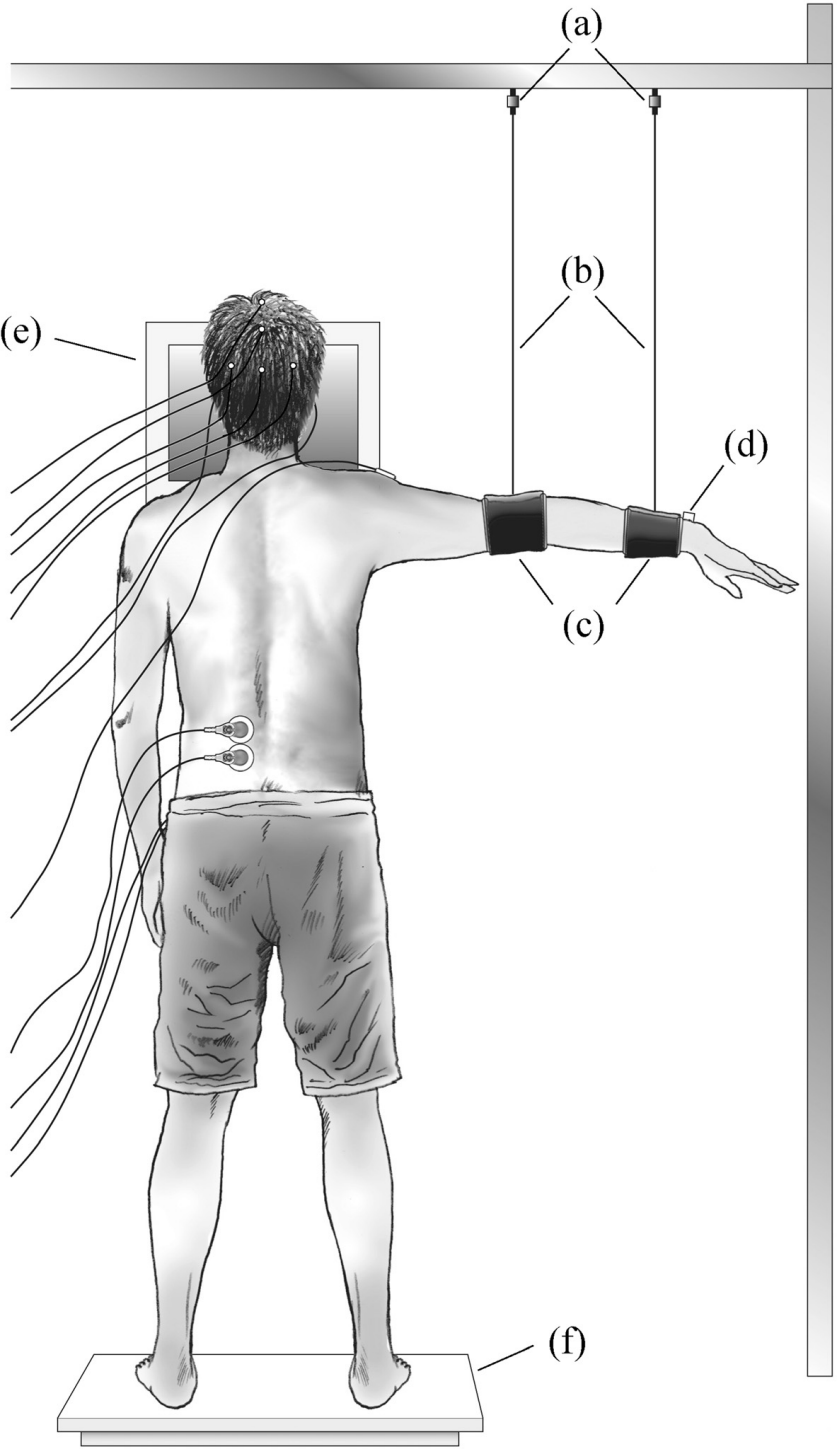
**Schematic of the experimental set-up in the shoulder abduction trials. (a)** Load cells; **(b)** metal wires; **(c)** bands; **(d)** accelerometer; **(e)** display; **(f)** force platform.

A force platform (5907044; Patella, Japan) was used to measure the position of the center of foot pressure in the mediolateral and anteroposterior directions (CoPml and CoPap, respectively) and the vertical component of the ground reaction force (Figure 1). To detect the start of shoulder abduction, a miniature unidirectional accelerometer (AS-5GB; Kyowa, Japan) was fixed on the dorsal surface of the right wrist, with the axis along a vertical line and the positive direction upward. A display was centrally placed 63 cm in front of the subject at eye height (Figure 1). Visual stimuli were presented on the display using the Multi Trigger System (MB72; Medical Try Systems, Japan).

To manipulate the extent of overlap between arm movement and appearance of the next S1, a suspended arm position was adopted as the initial arm position and total arm movement time was about 1.0 s. To suspend the right elbow and wrist, a metal frame was set outside the force platform with two bands and non-extensible metal wires (length: 70 cm; Figure 1). One band was placed around the wrist and the other band was placed around the elbow. The height of the frame was adjusted so that the initial height of the wrist was 10 cm below shoulder height with the elbow extended. The force applied to the bands was measured by two load cells (LUR-A-50NSA1; Kyowa, Japan) attached to each connection between the frame and wire. The total weight of the wristband was set at 1.5% of body weight using free weights in order to induce clear preceding activation of postural muscles [14].

Ag/AgCl cup electrodes (diameter: 8 mm, SEE203; GE Healthcare, Japan) for electroencephalogram (EEG) recording were affixed to the scalp at Fz, Cz, Pz, Oz, OL (halfway between O1 and T5), and OR (halfway between O2 and T6) in accordance with the international 10-20 system, and referred to the linked earlobe. A ground electrode was placed at Fpz. Horizontal and vertical electrooculograms (EOGs) were recorded in bipolar fashion from electrodes on the outer canthi of both eyes and electrodes above and below the left eye, respectively.

Ag/AgCl surface electrodes (diameter: 30 mm, P-00-S; Ambu, Denmark) were used in a bipolar derivation to record electromyographic (EMG) activity of the right middle deltoid (MD) as a focal muscle of shoulder abduction and the left erector spinae (ES) and gluteus medius (GM) as postural muscles [24]. The electrodes were aligned along the long axis of the muscle with an inter-electrode distance of about 3 cm.

Electrode input impedance was reduced to below 5 kΩ. Signals from electrodes were amplified (EEG: ×25,000; EOG and EMG: ×2,500) and band-pass-filtered (EEG: 0.05 to 100 Hz; EOG: 0.05 to 30 Hz; EMG: 5 to 1,000 Hz) using an amplifier (MA1132; Degitex, Japan). All electrical signals were sent to two separate computers for on-line EEG averaging (Dimension 1100; Dell Japan, Japan) and analysis (D530, Dell Japan, Japan) via A/D converters (ADA16-32/2(CB)F, CONTEC; Japan) with a 2,000-Hz sampling rate and 16-bit resolution.

### Visual stimuli

**Figure 2:**
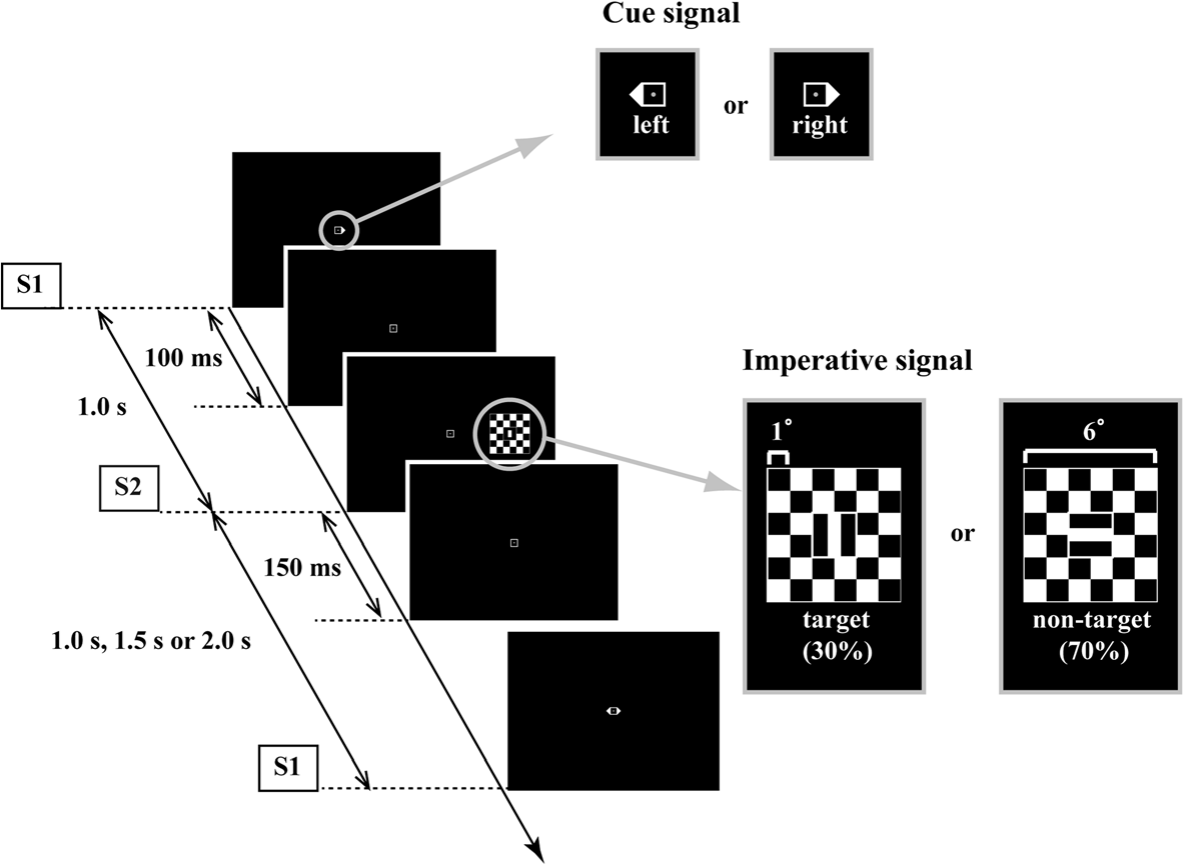
**Schematic of the visual stimuli (repeated S1-S2 sequence).** The imperative signal (S2) was presented at 9° to the left or right of the fixation point. The interval between S2 and the next cue signal (S1) was 1.0, 1.5, or 2.0 s. The S2-S1 interval was set as constant within each condition and changed among conditions to manipulate the time pressure. Within each block, the presentation of stimuli to the right and left of the fixation point and the presentation of target and non-target S2 stimuli were random.

Figure 2 shows the protocol for the presentation of visual stimuli, including the S1-S2 sequence. S1 was a triangle presented on either the left or right side of the central fixation point (1 × 1°). S2 was a checkerboard (6 × 6°) presented at 9° to the left or right of the fixation point (center to center). This is the outermost position where S2 is not in the blind spot [25]. S2 with a vertically oriented rectangle (0.5 × 2°) in the center was a target stimulus (probability of presentation: 30%) and S2 with a horizontally oriented rectangle in the center was a non-target stimulus (70% probability). Subjects were instructed to abduct the shoulder only in response to the target S2. S1 and S2 were presented for 100 ms and 150 ms, respectively, with 1.0-s interval. S2 was always presented at the position indicated by S1; thus, subjects could covertly focus attention on this position. This S1-S2 sequence was repeated for 100 s in each experimental block. The time interval from S2 to the subsequent S1 was 1.0, 1.5, or 2.0 s (defined as the 1.0-s, 1.5-s, and 2.0-s condition, respectively). The block number was different in each condition, as mentioned in the Procedure section. If the S2-S1 interval is constant in each condition, the timing of S1 following S2 is predictable [26]. Therefore, the S2-S1 interval was set as constant within each condition and changed among conditions to manipulate the time pressure. Within each block, the presentation of stimuli to the right and left of the fixation point and the presentation of target and non-target S2 stimuli were random.

### Procedure

All measurements were taken while subjects were standing barefoot with feet 27 cm apart and parallel on the force platform, and gazing at the fixation point (Figure 1). Initially, CoPml and CoPap positions were measured for 10 s while subjects maintained a quiet standing posture (QSP) with their arms by the side of their body. A total of five measurements were taken, with a 30-s period of seated rest between them. The means of the five measurements were used as the subject’s representative CoPml and CoPap positions during QSP.

Next, shoulder abduction trials were commenced with the right arm suspended (Figure 1). Subjects were instructed to keep their shoulder muscles as relaxed as possible and not to lean towards the bands. The absence of MD activity and force on the bands were monitored in real-time by an experimenter. Subjects maintained the CoPml and CoPap positions within ±1 cm of the position recorded in QSP [27] for at least 3 s, and then the presentation of visual stimuli began. In response to the target S2, regardless of the presentation position, subjects abducted their right arm by 10 cm at maximum speed, stopped voluntarily in a horizontal position, and maintained this position for a while before returning to the start position. Total arm movement time was about 1.0 s.

EEG waveforms for target and non-target S2 were averaged online using software (EPLYZER II; Kissei Comtec, Japan). Each block consisted of 50, 40, or 33 S2 stimuli in the 1.0-, 1.5- and 2.0-s conditions, respectively. The experimental blocks were repeated until the sufficient number of acceptable trials was obtained for each condition. For each side of S2 presentation, the acceptable number of target stimuli trials was more than 20 and the acceptable total number of target and non-target stimuli trials was more than 60. The criteria for acceptable trials were the following: (1) no eye movement (horizontal EOG below 0.5°); (2) no eye blink (vertical EOG voltage not exceeding ±100 μV); and (3) no excessive muscle-related potential (EEG voltage not exceeding ±100 μV at any electrode) during the period from 100 ms before to 800 ms after S2; as well as (4) CoP positions within ±1 cm of the QSP position just before trials that involved a shoulder abduction movement. Subjects sufficiently practiced each condition before the experimental trials to exclude the effects of habituation or novelty. Subjects had 30-s standing rest between each block, and 3-min seated rest between every two blocks and between conditions. The order of conditions was randomized for each subject. There was no effect of fatigue, as was confirmed by comparison of the reaction time and EMG amplitude of MD between first and last 10 trials in each condition.

### Data analysis

Data analyses were performed using BIMUTAS software (BIMUTAS II; Kissei Comtec, Japan). The attentional effect on the P1 and N1 components of the ERP is evident in ERPs recorded by occipital electrodes contralateral to the visual field of stimulus presentation, and is clearly elicited by stimuli in the right visual field [28,29]. This may be because both parietal association areas are activated when attending to stimuli in the right visual field, but only the right parietal association area is activated when attending to stimuli in the left visual field [30]. Thus, in the present study, to investigate the relation between each ERP component and postural muscle activation, analyses were performed only on trials in which the stimulus was presented in the right visual field. Trials where subjects did not respond correctly to S2 were defined as error trials, and were eliminated from the analysis. The percentage of error trials in each condition was calculated as the error rate.

#### ERPs

ERPs were averaged separately for each condition. The mean amplitude in the 100-ms period before S2 was defined as the baseline. The EEG epoch was from 100 ms before to 800 ms after S2.

For P1 and N1, waveforms from the OL electrode elicited by both target and non-target S2s were averaged [31-33]. The averaged waveform was smoothed using a 60-Hz low-pass filter. The largest positive peak from 80 to 130 ms and the largest negative peak from 140 to 220 ms after S2 were defined as P1 and N1, respectively. The peak-to-peak amplitude (P1-N1 amplitude) and the latencies of these peaks from S2 were calculated.

**Figure 3:**
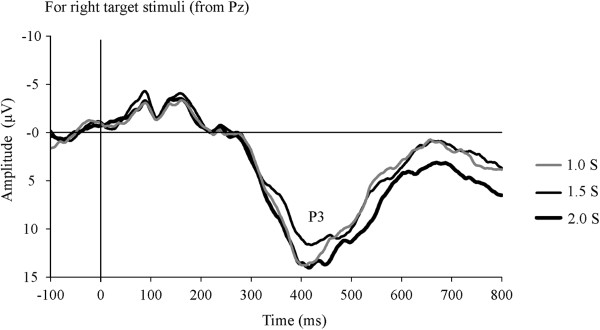
Grand average P3 waveform for target stimuli presented on the right of the fixation point.

N2 and P3 are elicited by target stimuli in a discrimination task [13,34]. For visual target stimuli, N2 and P3 are maximal in the occipital and parietal areas, respectively [9]. Therefore, these components were analyzed using EEG waveforms from the Oz and Pz electrodes elicited by target S2. The averaged waveform was smoothed using a 30-Hz low-pass filter because the P3 wave has a relatively low frequency (3 Hz) [6,35]. The largest negative peak between 200 and 350 ms after the target S2 was defined as N2. The amplitude of the peak relative to baseline and the latency of the peak from S2 were calculated.In many P3 waveforms, bimodal peaks were observed with about a 50-ms inter-peak interval (Figure 3). Accordingly, P3 was analyzed as follows: first, the largest positive peak between 250 and 600 ms after the target S2 was defined as P3. A second P3 peak was defined as present when the second-largest positive peak observed from -100 ms to +100 ms of the largest P3 peak was larger than 70% of the largest peak amplitude, and when the amplitude of the trough between these two positive peaks was less than 95% of the second-largest peak amplitude. These P3 peaks were referred to as the first and second peaks according to the latency. The amplitude of the first P3 peak relative to baseline and the latency of the peak from S2 were calculated. The slopes of the regression line of P3 waveform were calculated for the 100 ms before and after the first P3 peak. Furthermore, the percentage of subjects with bimodal P3 peaks was calculated in each condition.

#### EMG

**Figure 4:**
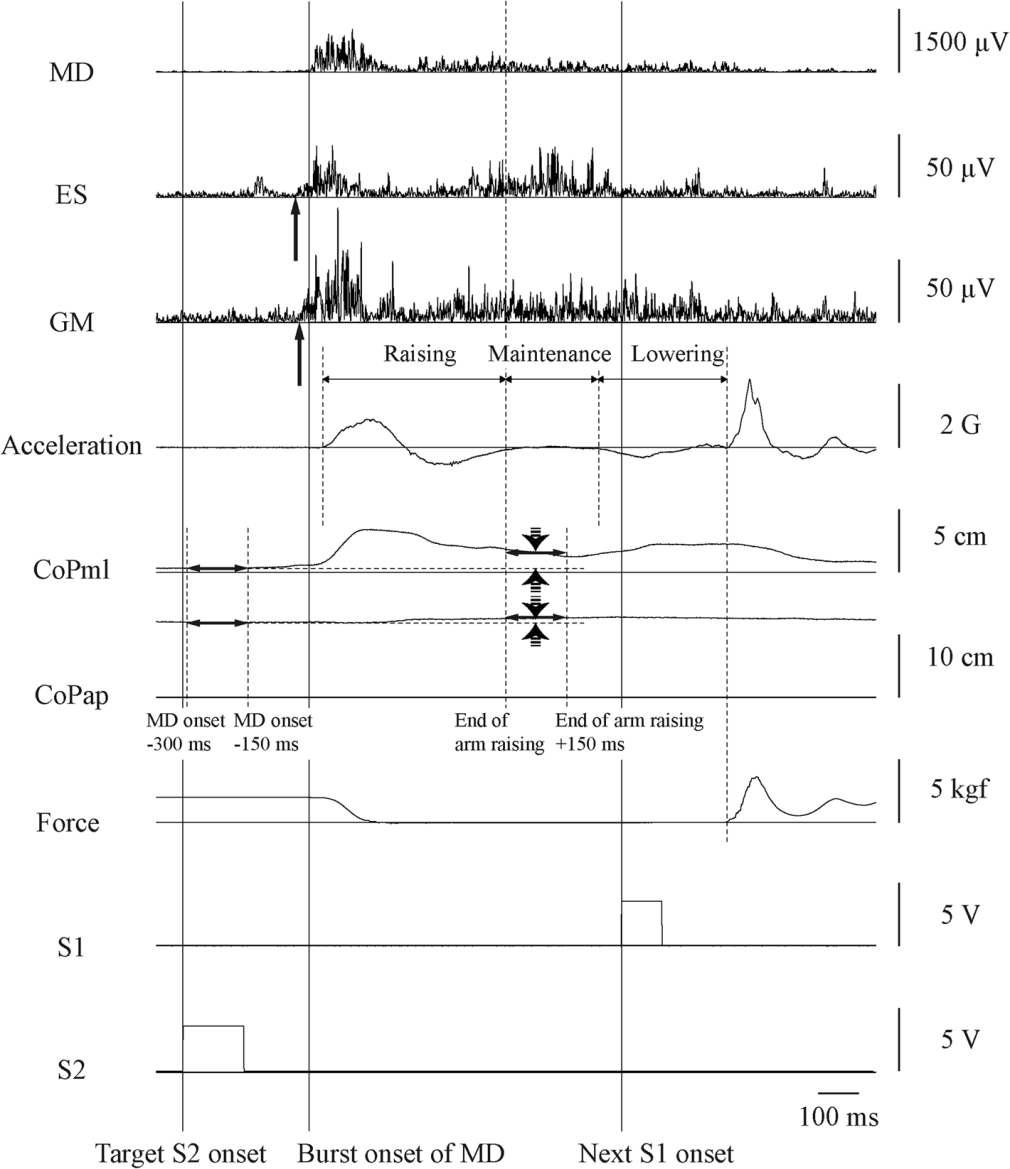
**Representative waveforms during shoulder abduction in the 1.0-s condition.** MD: right middle deltoid; ES: left erector spinae; GM: left gluteus medius; Acceleration: arm movement acceleration; CoPml: center of pressure in the mediolateral direction; CoPap: center of pressure in the anteroposterior direction; Force: force applied to the wristband; S1: cue signal; S2: imperative signal. Solid vertical arrows indicate ES and GM burst onset. Dashed arrows indicate CoPml and CoPap displacement.

EMG data were analyzed as described below, with reference to a previous study [36] (Figure 4). To exclude electrocardiographic and movement artifacts, all EMG data were high-pass filtered at 40 Hz using a seventh-order Butterworth method and then full-wave rectified. Background MD activity was calculated during the 150 ms prior to a target S2. Burst activation of MD was identified when the amplitude of the envelope line increased more than two SDs above the mean of the background activity for at least 50 ms and when the onset of this increase occurred 200 to 500 ms after target S2 onset. MD reaction time was defined as the time from target S2 onset to MD burst onset. Background ES and GM activity was calculated from 300 to 150 ms prior to MD burst onset. Burst activation of postural muscles was identified when the amplitude of the envelope line increased more than two SDs above the mean of the background activity for at least 50 ms, and when the onset of this increase occurred between 150 ms prior to and 100 ms after MD burst onset. The onset time of postural muscle activity was expressed relative to MD burst onset, and is presented as a negative value when burst onset of postural muscles preceded MD burst onset.

To analyze the magnitude of activity of each muscle, the rectified EMG from -100 to +200 ms with respect to burst onset was averaged for all acceptable trials in each condition. The averaged EMG waveforms were smoothed using a 40-Hz low-pass filter and the peak amplitude was measured relative to baseline.

#### Duration of arm movement

The first positive deviation in the accelerometer signal was defined as the start of shoulder abduction (arm raising; Figure 4). The end of arm raising was the point at which the second burst of MD activity dropped below the mean + two SDs for 200 ms just before arm lowering. The maximum vertical force measured by the force platform was referred to determine this point. The start of arm lowering was defined as the first negative deviation in the accelerometer signal after the end of arm raising. The end of arm lowering was defined as the point at which the force applied to the wristband started to increase. The durations between the start and end of arm raising, maintenance and lowering were calculated. The offset time of arm lowering was calculated as the duration from the end of arm lowering to the onset of the next S1. This is presented as a negative value when the end of arm lowering preceded the next S1.

#### CoP

Mean CoPml and CoPap positions were calculated from 300 ms to 150 ms prior to first burst of MD activation (before arm movement) and during the 150 ms after the end of arm raising (Figure 4). The differences between these mean positions were considered CoPml and CoPap displacements.

### Statistical analysis

Shapiro-Wilk tests confirmed that all data satisfied the assumption of a normal distribution. A one-sample *t*-test was used to assess whether the offset of arm lowering significantly differed from onset of the next S1. Cochran’s Q-test was used to compare the percentage of subjects with bimodal P3 peaks across conditions. For other parameters, one-way repeated-measures analysis of variance (ANOVA) was used to assess the effect of condition. Post-hoc multiple comparisons were performed using Tukey’s honestly significant difference test to further examine significant differences suggested by ANOVA. In order to evaluate the correlation between parameters, each variable was normalized for each subject using the following formula:

$$\begin{array}{ll} \;\mathrm{Standard}\;\mathrm{score}\left(Z\right)= & \left(\mathrm{Value}\;\mathrm{in}\;\mathrm{one}\;\mathrm{condition}-\mathrm{mean}\;\mathrm{across}\;\mathrm{all}\;\mathrm{conditions}\right)/\\ & \mathrm{SD}\;\mathrm{across}\;\mathrm{all}\;\mathrm{conditions} \end{array}$$

The correlations between these standard scores were then evaluated using Pearson correlation coefficients. The alpha level was set at *P* <0.05. All statistical analyses were performed using SPSS (14.0 J; SPSS Japan, Japan).

## Results

**Figure 5:**
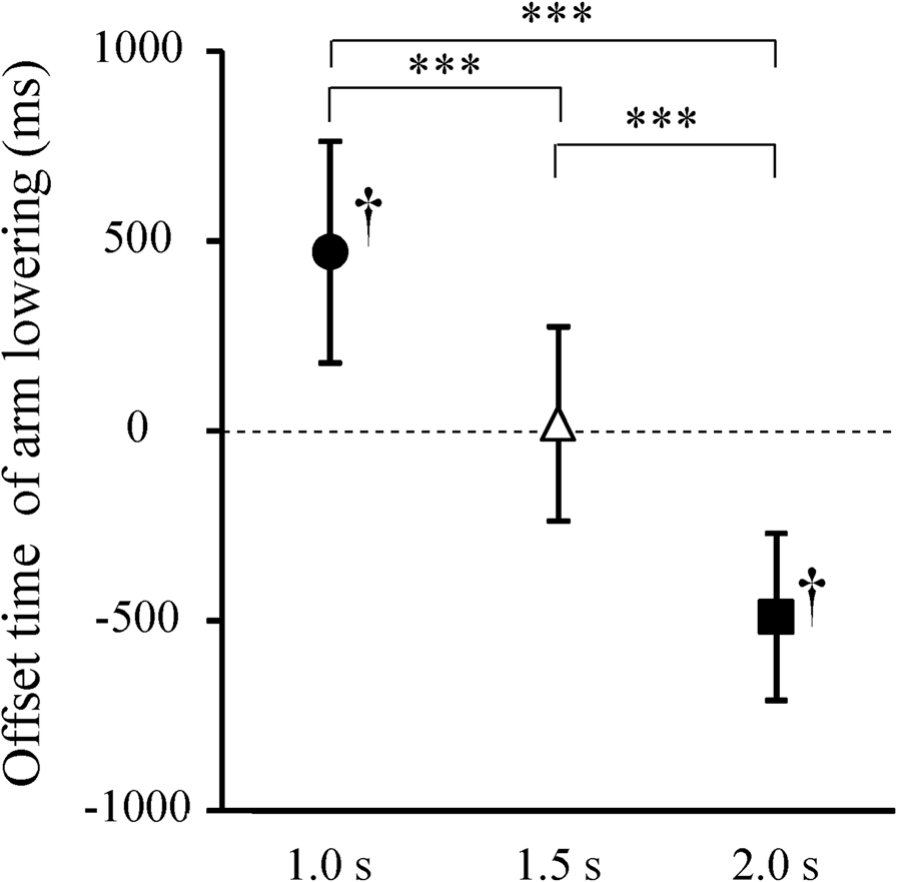
**Means and standard deviations of the offset time of arm lowering.** Time zero (horizontal dashed line) indicates onset of the next cue signal (S1). Negative values indicate that the arm lowering ended before the next S1. † indicates significantly different from zero (*P* <0.001). ***indicates significant difference between conditions (*P* <0.001).

There was no significant effect of condition on the duration of arm raising, maintenance, or lowering (Table 1). The mean (SD) of the offset time of arm lowering was 471.7 (292.3), 17.9 (256.3), and -490.8 (220.0) ms in the 1.0-, 1.5-, and 2.0-s conditions, respectively, and there was a significant effect of condition (*F*_1,17_ = 306.5, *P* <0.001; Figure 5). The offset time significantly increased as S2-S1 interval decreased (all post-hoc *P* <0.001). The offset time was significantly earlier than the next S1 onset in the 2.0-s condition and significantly later in the 1.0-s condition (both *P* <0.001). There was no significant effect of condition on the mean CoPml and CoPap positions before arm movement or the CoPml and CoPap displacements (Table 1).

**Table 1:** Means and standard deviations (SD) of data related to motor output

**Dependent variable**	**Condition**	**Mean ± SD**	**Statistical values of one-way ANOVA**	**Significance**
Duration of arm raising (ms)	1.0 s	457.6 ± 52.7	*F*_*2,24*_*= 1.4*	N.S
	1.5 s	478.0 ± 74.2		
	2.0 s	477.1 ± 82.3		
Duration of arm maintenance (ms)	1.0 s	304.1 ± 245.9	*F*_*1,15*_*= 0.02*	N.S
	1.5 s	298.9 ± 203.0		
	2.0 s	301.5 ± 189.8		
Duration of arm lowering (ms)	1.0 s	361.1 ± 55.1	*F*_*2,24*_*= 0.1*	N.S
	1.5 s	365.5 ± 47.9		
	2.0 s	364.6 ± 47.3		
CoPml mean position before arm movement (cm)	1.0 s	0.2 ± 1.3	*F*_*2,24*_*= 0.7*	N.S
	1.5 s	0.2 ± 1.2		
	2.0 s	0.1 ± 1.1		
CoPml displacement (cm)	1.0 s	1.4 ± 0.6	*F*_*2,24*_*= 1.0*	N.S
	1.5 s	1.4 ± 0.7		
	2.0 s	1.5 ± 0.8		
CoPap mean position before arm movement (cm)	1.0 s	10.1 ± 1.6	*F*_*1,17*_*= 0.4*	N.S
	1.5 s	10.0 ± 1.1		
	2.0 s	10.2 ± 1.3		
CoPap displacement (cm)	1.0 s	0.8 ± 0.3	*F*_*2,24*_*= 0.5*	N.S
	1.5 s	0.7 ± 0.3		
	2.0 s	0.8 ± 0.3		
Error rate of response (%)	1.0 s	0.8 ± 1.0	*F*_*2,24*_*= 0.9*	N.S
	1.5 s	1.4 ± 2.6		
	2.0 s	0.9 ± 1.2		
MD reaction time (ms)	1.0 s	311.4 ± 39.6	*F*_*2,24*_*= 9.9*	*p* < 0.001
	1.5 s	335.5 ± 50.2^a^		
	2.0 s	333.0 ± 36.6^a^		
EMG peak amplitude of MD (μV)	1.0 s	293.5 ± 182.6	*F*_*2,24*_*= 1.41*	N.S
	1.5 s	287.5 ± 160.5		
	2.0 s	308.1 ± 185.3		
EMG peak amplitude of ES (μV)	1.0 s	23.5 ± 13.2	*F*_*2,24*_*= 3.03*	N.S
	1.5 s	19.9 ± 8.5		
	2.0 s	23.4 ± 11.5		
EMG peak amplitude of GM (μV)	1.0 s	29.3 ± 12.9	*F*_*2,24*_*= 0.14*	N.S
	1.5 s	30.1 ± 15.9		
	2.0 s	29.4 ± 11.0		

CoPmI: center of foot pressure in the mediolateral direction, CoPap: center of foot pressure in the anteroposterior direction, MD: middle deltoid, ES: erector spinae, GM: glueus medius, EMG: electromyogram, ANOVA: analysis of variance.

^a^indicates a significant differences compared to 1.0-s condition (*p < 0.01*).

There was no significant effect of condition on the amplitude of P1-N1 or N2, the latency of P1, N1, N2, or the first P3 peak, or the slope of the regression line for the 100 ms before the first P3 peak (Table 2). There was a significant effect of condition on the amplitude of the first P3 peak and the slope of the regression line for the 100 ms after the first P3 peak (amplitude: *F*_2,24_ = 4.8, slope: *F*_2,24_ = 4.4, both *P* <0.05; Table 2). The amplitude of the first P3 peak was significantly smaller in the 1.5-s condition than in the 1.0- and 2.0-s conditions (both *P* <0.05). The slope of the regression line for the 100 ms after the first P3 peak was significantly larger in the 1.0-s condition than in the 1.5- and 2.0-s conditions (both *P* <0.05). The percentage of subjects with bimodal P3 peaks was significantly smaller in the 1.0-s condition than in the 2.0-s condition (*Q*_1_ = 4.5, *P* <0.05; Table 2).

**Table 2:** Means and standard deviations (SD) of components of event-related potentials

**Dependent variable**	**Condition**	**Mean ± SD**	**Statistical values of one-way ANOVA**	**Significance**
P1 latency (ms)	1.0 s	103.0 ± 10.4	*F*_*2,24*_*= 1.2*	N.S
	1.5 s	103.2 ± 11.4		
	2.0 s	102.1 ± 10.5		
N1 latency (ms)	1.0 s	156.6 ± 9.7	*F*_*2,24*_*= 1.2*	N.S
	1.5 s	157.7 ± 12.4		
	2.0 s	154.7 ± 12.4		
P1-N1 amplitude (μV)	1.0 s	8.2 ± 4.0	*F*_*2,24*_*= 0.2*	N.S
	1.5 s	8.3 ± 3.4		
	2.0 s	8.7 ± 3.8		
N2 latency (ms)	1.0 s	265.7 ± 26.7	*F*_*2,24*_*= 0.6*	N.S
	1.5 s	263.1 ± 26.3		
	2.0 s	265.8 ± 24.0		
N2 amplitude (μV)	1.0 s	3.4 ± 5.2	*F*_*2,24*_*= 0.8*	N.S
	1.5 s	3.4 ± 4.5		
	2.0 s	2.4 ± 4.1		
Latency of the first P3 peak (ms)	1.0 s	409.6 ± 27.8	*F*_*1,15*_*= 0.8*	N.S
	1.5 s	413.5 ± 41.3		
	2.0 s	407.7 ± 30.4		
Amplitude of the first P3 peak (μV)	1.0 s	15.8 ± 4.7	*F*_*2,24*_*= 4.8*	*p* <0.05
	1.5 s	13.5 ± 6.3^a^		
	2.0 s	15.8 ± 5.9^b^		
The slope of the P3 waveform in the 100 ms before the first peak	1.0 s	-0.13 ± 0.05	*F*_*2,24*_*= 2.4*	N.S
	1.5 s	-0.10 ± 0.04		
	2.0 s	-0.12 ± 0.05		
The slope of the P3 waveform in the 100 ms after the first peak	1.0 s	0.07 ± 0.04	*F*_*2,24*_*= 4.4*	*p* <0.05
	1.5 s	0.04 ± 0.07^a^		
	2.0 s	0.04 ± 0.03^a^		
The percentage of subjects with bimodal P3 peaks (%)	1.0 s	23.1	*Q*_*1*_*=4.5*	*p* <0.05
	1.5 s	46.2		
	2.0 s	69.2^a^		

ANOVA: analysis of variance.

^a^indicates a significant difference compared to 1.0-s condition (*p* <0.05).

^b^Indicates a significant difference compared to 1.5-s condition (*p* <0.05).

**Figure 6:**
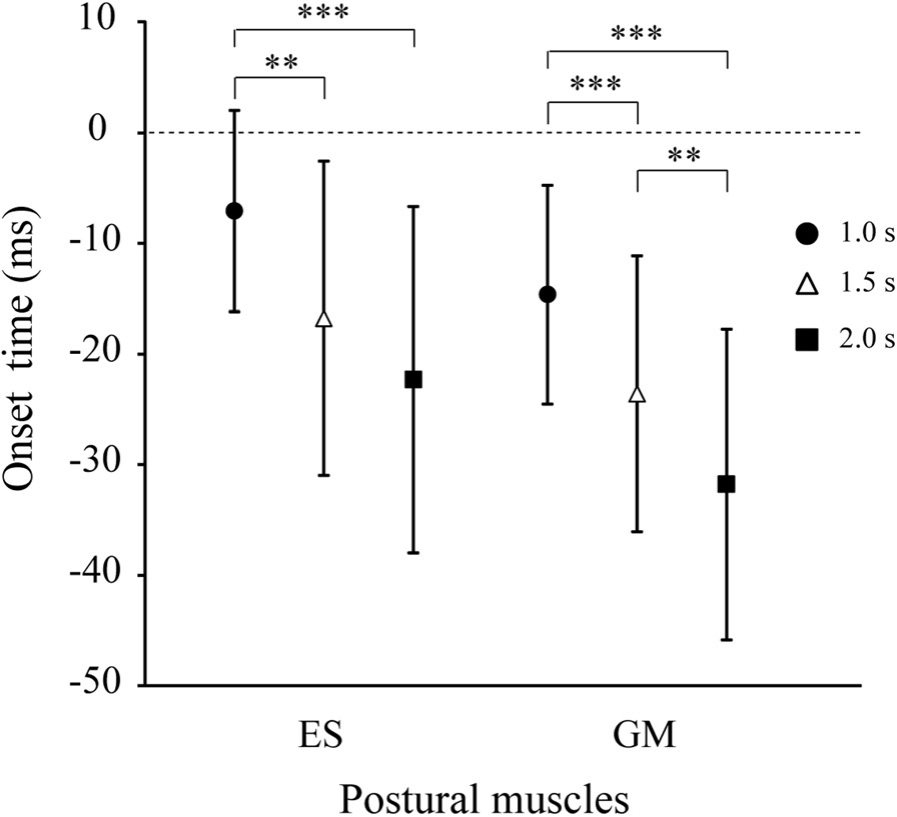
**Means and standard deviations of the onset time of postural muscles relative to the onset of the middle deltoid.** ES: left erector spinae; GM: left gluteus medius. Time zero (horizontal dashed line) indicates onset time of the middle deltoid. **indicates *P* <0.01 and ***indicates *P* <0.001 between conditions.

There was no significant effect of condition on the error rate (Table 1). There was a significant effect of condition on MD reaction time (*F*_2,24_ = 9.9, *P* <0.001; Table 1) and the onset times of ES and GM (ES: *F*_2,24_ = 18.5; GM: *F*_2,24_ = 24.4, both *P* <0.001; Figure 6). MD reaction time was significantly shorter in the 1.0-s condition than in the 1.5- and 2.0-s conditions (both *P* <0.01). The onset times of ES and GM were significantly later in the 1.0-s condition than in the 1.5- and 2.0-s conditions (all *P* <0.01). The onset time of GM was also significantly later in the 1.5-s condition than in the 2.0-s condition (*P* <0.01). There was no significant effect of condition on the EMG peak amplitude of MD, ES, or GM (Table 1).

MD reaction time was negatively correlated with the onset time of ES and GM (ES: *r* = -0.58; GM: *r* = -0.67, both *P* <0.001). The onset time of GM was positively correlated with onset time of ES (*r* = 0.80, *P* <0.001) and the slope of the waveform after the first P3 peak (*r* = 0.49, *P* <0.01). There were no other significant correlations between any variables.

## Discussion

The arm raising performed in response to target S2 ended before the onset of the next S1 in all conditions. The arm lowering ended about 470 ms after the onset of the next S1 in the 1.0-s condition, just at the S1 onset in the 1.5-s condition, and about 490 ms before the S1 onset in the 2.0-s condition. It takes about 400 ms after S1 onset to completely shift covert spatial attention to the indicated presentation position of S2 [37,38]. Thus, in the 1.0-s condition, the arm lowering and attentional shift from S1 to the next S2 would have been overlapped. This created a dual task where attention was divided between the arm motion and the attentional shift. Task performance in an oddball-like paradigm also requires careful attention to S1. Therefore, in the 1.0-s condition, the attentional shift from S2 to the next S1 would have been performed quickly, in order to reduce the above-mentioned overlap. By contrast, in the 2.0-s condition, the arm lowering did not overlap with the attentional shift from S1 to the next S2, and then participants had enough time for these two processing. In the following paragraphs, we discuss the effects of these time pressures on cognitive and behavior processing in more detail.

The arm raising started before the P3 peak in all conditions. The shoulder abduction was executed transiently and would have been controlled by open loop. Thus, in the present study, the execution of the motor program related to the arm movement would have ended before the P3 peak. P3 is considered to reflect cognitive processing, such as the evaluation and judgment of S2 [11] and the subsequent context updating including attentional shift [12,13]. In this study, there was no significant difference among conditions in the latency of the first P3 peak. However, the number of subjects with bimodal P3 peaks was less in the 1.0-s condition than in the 2.0-s condition, and the slope of the waveform after the first P3 peak was significantly steeper in the 1.0-s condition than in the other two conditions. These results are consistent with a previous study that investigated the effect of time pressure on P3 [17] and showed that the second P3 peak occurred soon after the first peak in the 1.0-s condition. This suggests that the speed of the latter half of cognitive processing, including attentional shift, became faster as time pressure increased. Similarly, MD reaction time was shorter in the 1.0-s condition than in the other conditions, as previous reports that reaction time is strongly affected by time pressure [3-5].

On the other hand, the amplitude of the first P3 peak had a U-shaped relation with S2-S1 interval, being significantly larger in the 1.0-s and the 2.0-s condition than in the 1.5-s condition. P1-N1 and N2 amplitudes showed no significant difference among conditions, and these amplitudes had no differences with high amplitudes in the previous study where attention was focused on S2 [36]. These suggest that in the 1.0-s condition, the amplitude of the first P3 peak would reflect the higher attention directed to all the cognitive processing to shorten the processing time. In the 2.0-s condition, there was no overlap between the arm movement and attentional shift from S1 to S2, and participants had enough time to carry out the latter part of cognitive processing, allocating a lot of attention to this processing. The smaller P3 amplitude in the 1.5-s condition showed that the time pressure was intermediate among the three conditions.

There was no significant effect of time pressure on the magnitude of activity of any muscle or the CoPap and CoPml displacements, but the onset of postural muscle activity became later as the S2-S1 interval decreased. In the present study, motor preparation and anticipatory attention directed to S2 could not be evaluated using CNV [20] because of the overlap between the arm lowering and attentional shift from S1 to the next S2. However, CNV significantly correlates with the onset time of postural muscles with respect to the focal muscle [22,23]. Therefore, we used the onset time of postural muscles to evaluate the postural preparation associated with arm movement and anticipatory attention directed to S2. The onset time of GM was positively correlated with P3 slope. Thus, as the time pressure increased, hastening the cognitive processing including the attentional shift would lead to insufficient postural preparation associated with arm movement and anticipatory attention directed to S2.

## Conclusions

The findings of this study suggest that with a shorter S2-S1 interval, that is, higher time pressure, attention was allocated to hasten the latter part of cognitive processing that may relate to attentional shift from S2 to next S1, which led to insufficient postural preparation associated with arm movement and anticipatory attention directed to S2.

The present study adds new knowledge of the effects of time pressure on cognitive processing, including attentional shift, and anticipatory postural control during repeated task performance. Using the methods developed in this study, we can conduct studies that investigate the effect of time pressure on cognitive processing and anticipatory postural control in tasks with behavior processing of varying difficulty or multiple cognitive tasks.

## Abbreviations

ANOVA: Analysis of variance; CNV: Contingent negative variation; CoP: Center of foot pressure; CoPap: Center of foot pressure in the anteroposterior direction; CoPml: Center of foot pressure in the mediolateral direction; EEG: Electroencephalogram; EMG: Electromyography; EOG: Electrooculogram; ERP: Event-related potential; ES: Erector spinae; GM: Gluteus medius; MD: Middle deltoid; QSP: Quiet standing posture; S1: Cue signal; S2: Imperative signal; S2-S1 interval: Interval from the onset of the imperative signal to the onset of the next cue signal; SD: Standard deviation.

## Competing interests

The authors declare that they have no competing interests.

## Authors’ contributions

The contribution of each author is as follows: KA and KF developed the idea for the study, planned the methods, directed the experiments, interpreted the results, and drafted the manuscript. CY and NK contributed to the experiments, data analysis, and manuscript preparation. All authors read and approved the final manuscript.
